# On the Medical Properties of the Piscidia Erythrina, or Jamaica Dogwood

**Published:** 1832-09

**Authors:** William Hamilton

**Affiliations:** Corresponding Member of the Medico-Botanical Society.


THE LONDON
### Medical and Physical Journal.

403, VOL. LXVIII.] SEPTEMBER 1832. [75, *New Series*.

"For many fortunate discoveries in medicine, and for the detection of numerous errors, the world is indebted to the rapid circulation of Monthly Journals; and there never existed any work to which the Faculty, in Europe and America, were under deeper obligations than to the 'Medical and Physical Journal of London, now forming a long but invaluable series."—RUSH.

### ORIGINAL PAPERS.

TRANSACTIONS OF THE MEDICO-BOTANICAL SOCIETY OF LONDON,
*Published under the Direction of the President and Council.*

### JAMAICA DOGWOOD.

### On the Medical Properties of the Piscidia Erythrina, or Jamaica Dogwood. By William Hamilton, m.b., Corresponding Member of the Medico-Botanical Society.

The Jamaica dogwood tree, or Piscidia Erythrina, is a small branching tree, of from fifteen to twenty feet in height, common in the low grounds near the sea, in most of the West India islands, and every where by the road sides (according to Jacquin) in Jamaica, where it flowers, according to my observations, in the months of March, April, and May, during which it is wholly destitute of leaves, which rarely appear before the period of inflorescence has passed. It belongs to the Linnæan class and order of Diadelphia Decandria, and is distinguished from other plants of the same class and order by its acute stigma, and four-winged legume, enclosing a number of compressed, oblongo-reniform seeds. Its leaves, which are periodically deciduous, are unequally pinnated, with ovate, very entire, pubescent leaflets. Towards the middle or latter end of March, thyrsoidal racemes of white papilionaceous flowers, of rather a large size, wholly destitute of smell, make their appearance at the extremities of the younger branches, and continue progressively expanding till about the middle of May, when they are succeeded by clusters of linear compressed legumes, furnished with four membranaceous, longitudinal wings, greatly exceeding the legume itself in breadth: the legume consists of one cell, nearly united between the seeds, so as to appear to a careless observer like a many-celled legume. The seeds, which I have always observed to be very much compressed, and of an oblong reniform shape, Swartz describes as roundish.

According to Jacquin, the leaves and branches of this tree, bruised and mixed with water, intoxicate the fish it contains, making them swim blindly on the surface, so as to become an easy prey to the fisherman: his words are, "Folia ramulique contusa, et aquis injecta, pisces inebriant, ut aquis supernatent, manuque capi possint: quam virtutem cum multis aliis plantis Americanis communem hæc arbor possidet." Among the other West Indian plants to which he refers in the concluding part of the sentence as sharing this property of intoxicating fish, are the Jacquinia Armillaris, called by the Spaniards *el Barbasco*, by the French *Bois bracelets*, and by the English *piecrust*, a low but ornamental shrub common on the seacoast in most of the Antilles, and an ingredient in one of the most deadly of the toxiques of South America; the Galega toxicaria, and a plant of which I have never been able to obtain any true account, which the Carribs of St. Vincent were said to employ, in a somewhat different manner, for the same purpose, under the name of *Wonga root*. From the similarity of the effect produced by all these various substances on the animal economy, it is not unreasonable to conjecture that this uniformity of action arises from the uniform presence of the same active principle in each, analogous to the morphine, quinine, tannin, &c., which are found to pervade a variety of dissimilar plants, communicating to them, however, a similarity of properties more or less decided according to the degree of concentration in which it exists in each. Hence it might be worth while to subject them all to the test of medical experiment, in order to determine how far their active properties are capable of being rendered subservient to the wants of mankind.

In the only instance in which I have had an opportunity of witnessing the powerful effect of the dogwood as auxiliary to the fisherman, it was the bark of the roots, and not the leaves and young branches, which were employed. This bark was gathered at the season of flowering, and at the lunar period at which, according to the well-ascertained law of intratropical vegetation, the juices of the plant were in the highest state of activity, namely, the full moon in April. To those whose acquaintance with the phenomena of vegetable life is limited to the temperate zone, the idea of lunar influence upon the circulation of the sap may appear more visionary than correct; but to those who have resided for any time within the tropics, the fact is perfectly familiar, and the importance of strict attention to it fully known: the influence of the lunar phases on the sap in trees is such as to render the strictest attention to the state of the moon at the time of felling timber necessary, if the timber is desired to be durable. The West Indies abound in the most valuable hard woods, such as the Zygophyllum arboreum, or Guayacan tree, which, if cut between the full and new moon, when the sap is dormant or descending, are almost indestructible; but which, if felled between the new and full moon, when the sap has begun to mount, will infallibly begin to decay before the expiration of ten years, or even a shorter period. Even the fact alluded to by the Roman poet of the influence of the phases of the moon on animal life, where he says,

"Lubrica nascentes implent conchylia Lunæ,"

is perfectly familiar to the West India planters, who never admit the Echini, or sea eggs, a well-known West Indian delicacy, to their tables, except when the moon is at the full, experience having taught them that this delicate shellfish improves with the increasing, and goes out of season with the waning moon. This digression may appear foreign to my purpose; but will, I trust, appear excusable when I state that, from the disappointments I have myself experienced from neglecting a due attention to what I was at first disposed to regard as a vulgar error, I have been fully convinced that the activity or inertness of the bark of the Piscidia depends as much upon the season of the year and the period of the moon at which it has been gathered, as that of digitalis, colchicum, or any of the other valuable remedies with which the Flora of Britain presents us.

The bark of the dogwood root, collected at the period I have stated, previous to being used for fish poisoning, as the sport is called, is macerated with the lees of the stillhouse, and temper or quicklime; and put into baskets of a convenient size, with one of which each of the fishermen is provided: thus equipped, one or more of them embark in one or more boats, according to the size of the bay selected for the sport, and, pushing to a sufficient distance from the shore, they hold their baskets over the side of the boat in the water, which they continue to agitate with their baskets till the whole of their contents is washed out, and the water has become impregnated with the intoxicating preparation, which happens sooner and to a wider or narrower extent according to the number of washers and boats, and the dimensions of the bay. In a little time the smaller fish are seen floating, apparently dead, upon the surface of the water, while the larger fish, capable of longer resisting the stupifying influence of the medicated water, swim wildly about, raising their heads above the narcotic fluid, and striving, as it were, to breathe a purer atmosphere: these surrender themselves an easy prey to the persons in the boats, who catch them with their hands as they float by, perfectly unresisting; if thrown, immediately after being taken, into fresh and pure seawater, there is no doubt but that, with the exception perhaps of the smaller fry, they would soon recover. Neither their flavour nor wholesomeness is in the least impaired by the manner in which they have been taken; but, from the number which are uselessly destroyed by this mode of taking fish, poisoning has been prohibited in many of our islands. The manner in which the Wonga root was used by the Carribs differs in appearance from this, which I myself witnessed, but in principle is indisputably the same: they stuffed, as I was informed, the bellies of several small fish with a preparation of the root, and threw the fish thus doctored overboard, when they were devoured with avidity by the larger fish: these latter being stupified by the dose, became, in their turn, the prey of the icthyophagists in the boats.

Struck with the singular and decided effect of the dogwood bark upon the fish, I was induced to investigate its properties as an internal remedy upon the human frame; and commenced, accordingly, a series of experiments upon myself with the bark, in substance, in infusion, in decoction, and in tincture; which last I found to be the only efficient and practicable mode of exhibition, since the active constituent appears to be a resin insoluble in any thing but rectified spirit: hence the necessity of the stillhouse lees, which contain alcohol in a highly concentrated state, in combination with a powerful and deleterious empyreumatic oil, in the preparation of the bark for fish poisoning.

My tincture was prepared by macerating one ounce of the coarsely powdered bark in twelve ounces, by measure, of rectified spirit, which I had brought with me from England, for twenty-four hours, and straining. The tincture thus obtained was of a fine honey yellow, and appeared to be fully impregnated with the active principle of the bark: it had nothing striking or offensive in its taste or smell, but, on being dropped into water, it communicated to it an opaline or milky hue, evidently from the separation of a resin; for, on suffering some of the undiluted tincture to evaporate in a glass, the sides were encrusted with a white film of the resin which remained behind. Labouring at the time under a severe toothach, which seemed to set sleep at defiance, I took at bedtime a drachm measure of this tincture in a tumbler of cold water, and lay down, with the uncorked phial in the one hand and the empty glass in the other, to speculate upon the manner of its operation on the system. The dose was by no means disagreeable to take, nor was its action on the mouth and throat unpleasant, like that of the bark in substance, which irritated the fauces like the Daphne mezereum or the croton oil; but, soon after swallowing the dose, I became sensible of a burning sensation in the epigastric region, spreading rapidly to the surface, and terminating in a copious diaphoresis, in the midst of which I was surprised by a sleep so profound that I was utterly unconscious of existence from about eight o'clock at night till eight the following morning, when I awoke free from pain of every description, and found myself still grasping the uncorked phial in one hand, from which not a drop had been spilled, and the empty glass in the other. No unpleasant sensation followed, as is usually the case after opiates, from the exhibition of what was perhaps a needlessly large dose; nor did a friend, whom, though in perfect health, I persuaded to repeat my experiment in his own, suffer the slightest inconvenience from an equally full dose: his only observation was, that he never had slept so sound in his life as he did that night. I next tried its efficacy as a topical application in cases of carious teeth, introducing a pledget of cotton impregnated with the tincture into the cavity, and never knew an instance of a return of pain after this application. I was next desirous of comparing its effects upon animalculæ in water with those of the tincture of opium: for this purpose I took, in two separate wine-glasses, equal quantities by measure of water, filled with the lively young of the mosquito, adding to the water in one glass a sufficient number of drops of the Tinctura opii to stupify the animalculæ, which fell in a mass to the bottom; I then dropped into the other an equal number of drops of the Tinctura Piscidiæ, with a similar result. Next, taking the first glass, and carefully decanting the water without disturbing the insensible mass of animalculæ, I poured upon them fresh portions of pure water, previously filtered, in order to prevent confusion; upon which they revived, and swam about as actively as if nothing had happened. I treated those in the glass to which the dogwood tincture had been added, but without the slightest effect: the most frequently repeated affusions of pure water were not of the least avail; the animalculæ were truly dead, and thus furnished a conclusive proof of the superior potency of the dogwood over the opium tincture, in equal quantities. Experiments are yet wanting to determine the minimum doses requisite in both cases, and these it were much to be desired to have instituted by some medical practitioner resident in the West Indies; taking care, however, to employ bark gathered about the full moon in April, when the plant is in flower, and the best rectified spirits, or even pure alcohol, in his experiments. An inattention to these cautions will completely defeat the object of the experiments, and, in place of obtaining an active and valuable medicinal preparation, he will obtain one perfectly worthless and inoperative.

It were also to be wished that a quantity of the bark, gathered at the proper season, and carefully dried, might be sent to this country, accompanied by a bottle of the tincture prepared upon the spot, and carefully secured against the decomposing influence of light, heat, and air, for the purpose of enabling practitioners here to determine whether the medical virtues of the bark or the tincture are least impaired by keeping and transportation, and whether the Piscidia is an article adapted for supplying the place of the opium of the Levant in the pharmaceutical preparations of this country.

It might also be desirable to institute a set of comparative experiments upon the properties of the bark and leaves of the young branches, which Jacquin speaks of as being employed to intoxicate fish; as well as upon the bark both of the roots and branches of the Jacquinia armillaris, and other plants employed for a similar purpose. In order to increase the utility of these experiments, the substance operated upon should be gathered at different seasons of the year, at different states of the moon, and in different stages of the growth of the plant, accurately noting and distinguishing the various results.

It is also of importance to determine, by careful analysis, the uniformity or the dissimilarity of the principle upon which the medical properties of these various substances depends, the proportion in which it exists in each, and the circumstances under which it is to be found in the greatest abundance and highest perfection; as likewise the precautions requisite for its preservation from decomposition.

From not being sufficiently alive to all these particulars at the time, when a residence upon the spot afforded me an opportunity of prosecuting the investigation with success, my experiments have been incomplete, and their results unsatisfactory. A letter on the subject, which I published in Nicholson's Journal, in October 1812, contains several errors, which subsequent experience has enabled me partially to correct; and the supply of bark noticed in that letter as sent to Mr. Carlisle for experiment, having been gathered at an improper time (in the month of June), proved inert, as I myself experienced, from having prepared some tincture from a portion which I retained for the purpose, was no doubt the reason why I never heard from that gentleman on the subject.

As an object equally interesting in a philosophical and a medical point of view, and as tending to transfer a most lucrative branch of commerce from Turkey to our own possessions, the Piscidia Erythryna is well entitled to the attention of the Medico-Botanical Society, to whom I now resign its further investigation, after having detailed my own imperfect experiments, and their result.

---

